# A Positioning System Design Based on Tunnel Magnetoresistance Sensors for Rapid Zoom Optical Lens

**DOI:** 10.3390/s25061820

**Published:** 2025-03-14

**Authors:** Junqiang Gong, Dameng Liu, Jianbin Luo

**Affiliations:** 1 State Key Laboratory of Tribology in Advanced Equipment, Tsinghua University, Beijing 100084, China; junqiang@union-optech.com (J.G.); ldm@tsinghua.edu.cn (D.L.); 2 Union Optech Co., Ltd., Zhongshan 528463, China

**Keywords:** tunnel magnetoresistance (TMR) sensor, position sensor, magnetic grating, magnetic interference, voice coil motor

## Abstract

In response to the accurate positioning issue for high-speed moving lens groups in rapid zoom optical lenses with voice coil motors (VCMs), we demonstrate a positioning system design based on tunnel magnetoresistance sensors. The equivalent magnetic charge method and finite element method (FEM) simulations were utilized to compute the magnetic field distribution of the magnetic grating encoder. Based on analytical computation, the optimal air gap *δ*_S_ between the sensor and magnetic grating is determined to be *δ*_S_ = 0.15 mm, which balances magnetic flux density amplitude and total harmonic distortion. In addition, a simplified fitting model is proposed to reduce computational complexity. We quantify the magnetic interference of VCM through three-dimensional flux leakage mapping by FEM analysis, deriving an optimal sensor position *O*_S_, with a 24 mm *y*-offset and 20 mm *z*-offset relative to the VCM’s origin *O*_V_. The position error caused by interference remains below 5 μm with maximum deviations at trajectory endpoints of the moving group. The original signal output is processed and corrected, and eventually, the measured displacement exhibits a linear relationship with actual displacement. Our study provides a comprehensive framework for the design and optimization of magnetic positioning systems in optical applications with electromagnetic motors.

## 1. Introduction

Rapid zoom optical lenses [1] can switch fleetly from a short-focus state with wide-area surveillance to a long-focus state with high-resolution identification, which may show great potential in many fields such as security surveillance [2], intelligence vision [3], and high-speed photography [4]. For instance, in unmanned surveillance, faster zooming enables capturing fast-moving targets in wide areas and identifying them with high-resolution images, reducing the probability of losing sight of targets. However, high-magnification zoom optical lenses often face significant zoom delay issues, which are caused by long-stroke moving groups at relatively low speeds. Compared to liquid lenses [5] and Alvarez lenses [6], which are more suitable for small-bore compact systems, voice coil motors (VCMs) with high thrust, response speed, precision, and durability have emerged as the preferred actuation technology in rapid zoom optical systems [1,7,8]. Therefore, moving lens groups are driven directly by linear VCMs without transmission mechanisms, and the problem of accurate positioning for high-speed groups has followed.

Generally, linear displacement sensor techniques in zoom lenses can be roughly classified into the following two categories: optical encoder-based and magnetic encoder-based. While optical sensors are widely recognized for their high resolution and precision, their operational limitations, such as susceptibility to dust, debris, and vibrations, significantly constrain their reliability in harsh environments [9]. In contrast, magnetic positioning systems exhibit inherent advantages [10,11,12] in cost-effectiveness, enhanced robustness against environmental contaminants, flexible customization, and resistance to vibration and impact, making them increasingly preferred for applications demanding long-term operational stability. Therein, compared to magnetic encoders based on the Hall effect [13] and anisotropic magneto-resistance effect, tunnel magneto-resistance (TMR) sensors [14] have been emerging, with higher sensitivity, lower power consumption, a lower temperature coefficient of resistance, and smaller size. Therefore, TMR sensors are especially suitable for the compact design of rapid zoom optical lenses, whose working environment is always with electromagnetic interference, vibration, and low/high temperature.

In recent years, research on improving TMR positioning systems’ performance has attracted much interest. C. Lee et al. [15] demonstrated an integrated positioning module on a single substrate to simultaneously sense the incremental and absolute scale line. They made all sensors parallel to the magnetic scale surface on the same substrate in the lithography stage, and alignment error between sensors during the installation was totally excluded. X. Wang et al. [16] presented a displacement sensor based on the TMR effect with a resolution of 800 nm in millimeter-level operation range. They employed chip-level Au-In bonding to implement low-temperature assembly of the TMR devices and exploited the interpolation circuit and multi-bridge detection to enhance the sensor’s sensitivity and accuracy. J. Silva et al. [17] developed a three-channel application-specific integrated circuit (ASIC) for position encoder readout, and the ASIC can mitigate offsets up to approximately 1.3× common-mode voltage and amplify signals with a gain of 43.5 dB.

For magnetic positioning systems, the most important is to carefully analyze the magnetic field distribution of the magnetic encoder to determine the air gap between the sensor and encoder, which is the most significant structural parameter. For instance, in Y. B. Muna and C. Kuo’s work [18], the finite element method (FEM) analysis of the magnetic field for the magnetic encoder and TMR sensor gives an insight into the design of a relatively accurate magnetic encoder. They identified the best distance of 2 mm between the magnet and the TMR sensor and chose ceramic to reduce magnetic interference. Based on FEM results, S. Wang et al. [19] analyzed the position of the TMR sensor in the air gap of a permanent magnet synchronous motor. However, the magnetic field distribution of the magnetic encoder is not ideal generally, and external interference should be considered. In practical applications, there are lots of relevant studies on the interference effect of external factors on positioning accuracy. G. F. Close et al. presented a new method for multiphysics simulation of integrated magnetic sensors, allowing the joint modeling of kinematic, magnetostatic, and integrated circuit behavior within a signal-flow system model [20]. They presented a new sensor design for the accurate and robust measurement of linear displacement based on an exhaustive analysis of practical Ferrite magnets, and the sensor’s total error is 1%, including manufacturing tolerances, trimming accuracy, temperature, aging effects, and practical magnet constraints [21]. B. Chen et al. [22] investigated the effect of flattening a cracked medium on the positioning accuracy of a linear magnetic encoder and improved the accuracy by modification of the magnetic medium and flattening conditions. In order to eliminate the background magnetic field in the TMR sensor’s location, S. Wang et al. [23] utilized a double-layer parallel cables magnetizer and magnetic flux concentrating plates to further improve the displacement measurement sensitivity.

In addition, magnetic interference from electromagnetic motors on TMR sensors cannot be neglected. Differing from the common magnetic displacement sensor packaged with magnetic grating, S. Wang et al. [24] utilized TMR sensors to detect the periodic magnetic field of the permanent magnet linear synchronous motor directly to sense the displacement of the mover. From another perspective, when the TMR sensor is closed to electromagnetic motors, particularly for compact systems, magnetic field leakage of motors may interfere with signals. For VCM, external leakage [25,26,27] should be further considered carefully, while more works concentrate on the magnetic field distribution in the air gap [28,29], which is necessary for electromagnetic force computation. It is necessary to analyze the influence of magnetic interference on the positioning accuracy of TMR-based displacement sensors, as it provides critical insights for component arrangement.

In this work, to address the above challenges, we systematically analyze a high-precision positioning system comprising a TMR sensor and magnetic grating encoder for high-magnification rapid zoom optical systems integrated with VCMs. The total length of magnetic grating is determined by the stroke of VCM and multiple magnets model. The equivalent magnetic charge (EMC) method and FEM simulations are utilized to verify the accuracy of analytical computation of magnetic field distribution for magnetic grating. Based on analytical computation, the optimal air gap between the sensor and magnetic grating is determined to be *δ*_S_ = 0.15 mm, which balances magnetic flux density amplitude and total harmonic distortion. In addition, a simplified fitting model is further proposed to reduce computational complexity. Furthermore, we quantify VCM-induced interference through three-dimensional flux leakage mapping, deriving an optimal sensor position (24 mm y-offset, 20 mm z-offset). The position error caused by interference remains below 5 μm with maximum deviations occurring at the trajectory endpoints of the moving group. The original signal output is processed and corrected, and eventually, the measured displacement exhibits a linear relationship with the actual displacement, demonstrating the positioning system’s robustness and precision. Our study provides a comprehensive framework for the design and optimization of magnetic positioning systems in high-performance optical applications with electromagnetic motors.

## 2. System Components and Working Principles

**Figure 1:**
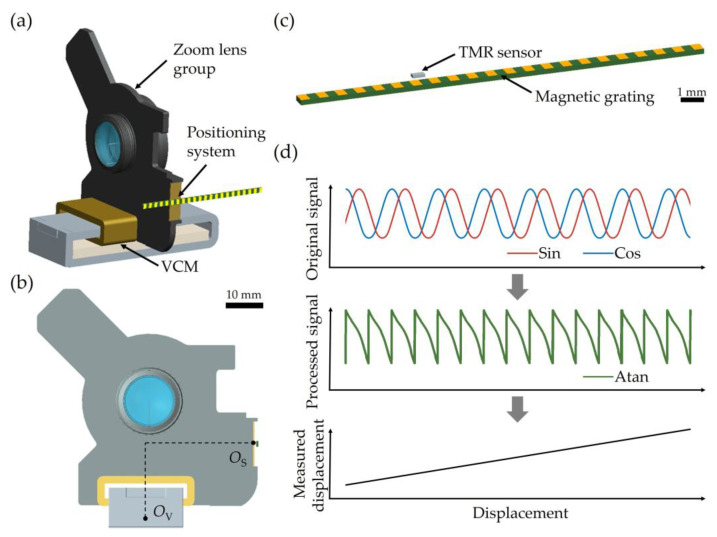
Schematic of core components of rapid zoom lens and sensing mechanism of positioning system. (**a**) Core components comprised of zoom lens group, VCM and positioning system. (**b**) Schematic of core components in the cross-sectional view. Scale bar: 10 mm. (**c**) Positioning system comprised of TMR sensor and magnetic grating. Scale bar: 1 mm. (**d**) Signal processing.

In a zoom optical system, rapid optical zoom is achieved through the high-speed relative movement of multiple lens groups, which are driven by VCMs. Figure 1a–c illustrates the schematic of the core components of the rapid zoom lens, designed using the structural design software Pro/ENGINEER 5.0. The positioning system consists of a TMR sensor and a magnetic grating encoder. The TMR sensor (TES2210-UCAB, TDK Co., Ltd., Tokyo, Japan) is moving with the moving group, which is fixed to the coil of the VCM. It is noteworthy that the sensor is mounted on a flexible printed circuit to enhance its long-term durability. The sensor is positioned above the top surface of the magnetic grating with a specific air gap *δ*_S_. As the stator, the magnetic grating generates a periodic magnetic field distribution. When the sensor moves with the zoom lens group, the relative motion and the TMR effect enable the sensor to detect the periodic magnetic flux density component parallel to the direction of motion, resulting in the generation of periodic signals corresponding to the displacement. The sensor comprises four TMR elements, forming a full Wheatstone bridge circuit. The full bridge circuit can be equivalent to two half-bridge circuits at the same fixed voltage, simultaneously generating two sinusoidal waveforms with a 90-degree phase difference (i.e., Sin and Cos). During signal processing, an arctangent (Atan) calculation is performed on these two waveforms to produce the processed signal output, Atan. After correction, the relative displacement can be derived from the processed signal, as shown in Figure 1d.

The positioning accuracy of the system is highly dependent on the clarity and precision of the waveform, which should exhibit minimal distortion. Therefore, it is critical to ensure that the magnetic field distribution sensed by the TMR sensor has adequate intensity and a sinusoidal profile. Accurate analytical computation of the magnetic field distribution generated by the magnetic grating is essential for determining the key parameter *δ*_S_. Additionally, magnetic interference from the VCM cannot be overlooked, and a thorough analysis of the VCM’s magnetic field is necessary to optimize the relative positioning between the sensor (*O*_S_) and the VCM (*O*_V_).

## 3. Analytical Computation of Magnetic Field Distribution for Magnetic Grating

### 3.1. Basic Model of EMC Method

**Figure 2:**
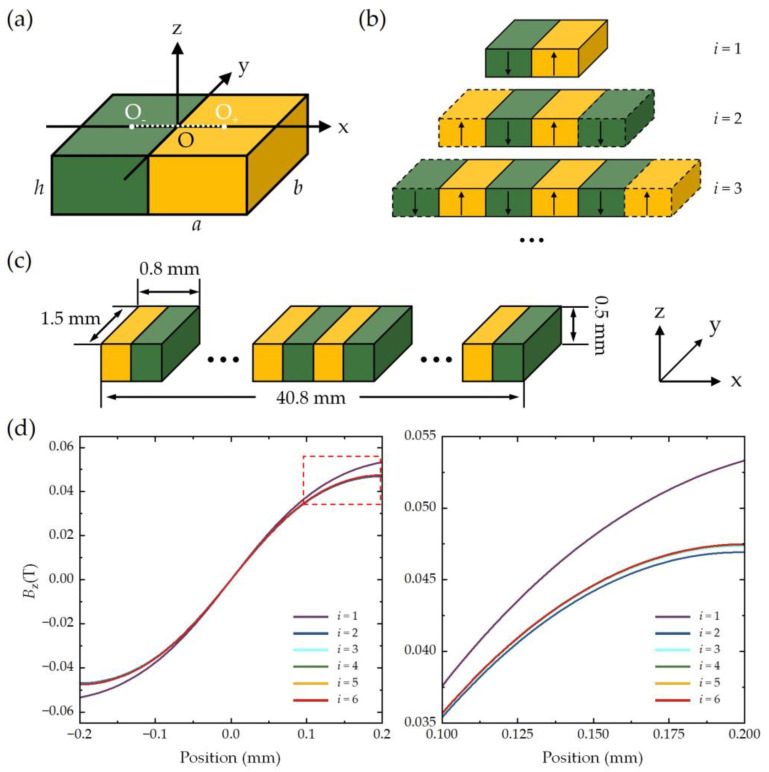
Schematic of different pairs (*i*) of magnets and corresponding magnetic flux density calculated by EMC method. (**a**) Schematic of one pair of magnets. (**b**) Schematic of more pairs of magnets. (**c**) Schematic of magnetic grating. (**d**) Calculated *B_z_* for different *i* at a certain air gap *δ*_S_ = 0.15 mm (y = 0). The right subfigure is partial enlarged view corresponding to red dashed box.

In order to analyze the optimal air gap *δ*_S_ between the TMR sensor and magnetic grating, an analytical computation of the magnetic field distribution generated by the magnetic grating is conducted. It is reasonable to assume that the magnetic grating consists of multiple pairs of rectangular bar permanent magnets, which are periodically arranged along the *x*-axis with alternating magnetization directions (along the *z*-axis), as illustrated in Figure 2a–c. The yellow magnets represent those polarized along the positive *z*-axis (denoted as ↑), and the green is for the opposite (↓). The magnetic field distribution above the magnetic grating, particularly within the spatial range corresponding to the center of a magnet pair (from O_−_ to O_+_), is of primary interest. This is because the magnetic field in the remaining regions can be derived from this segment (either identical or mirrored) when the number of magnets is sufficiently large. The number of magnet pairs is denoted as *i*. In the positioning system under study, the dimensions of the magnetic grating are specified as *a* = 0.4 mm, *b* = 1.5 mm, *h* = 0.5 mm, with a total length of 40.8 mm. Although there is a difference between the polarization distribution in reality and ideal assumption, the ideal, typical magnetic grating generates a somewhat similar field, especially for magnetic flux density amplitude calculation.

For a rectangular bar magnet, the analytical calculation of the magnetic field can be effectively conducted using the equivalent magnetic charge (EMC) method [30,31]. In this model, the magnet is conceptualized as a distribution of equivalent “magnetic charges.” Specifically, for a rectangular bar magnet with dimensions width *a*, depth *b*, and height *h*, polarized in the upward direction ↑, positive and negative magnetic charges are uniformly distributed on the top and bottom surfaces, respectively. The origin of the coordinate system is denoted as point O_+_, and the magnetic flux density components in the *x*, *y*, and *z* directions are represented as *B_x_*, *B_y_*, and *B_z_*, respectively. According to the EMC method, the components *B_x_*, *B_y_*, and *B_z_* originating from the positive magnetic charge are expressed as follows:

(1) $$B_{x+}=\frac{B_{r}}{4\pi }\left[\ln\frac{y-\frac{b}{2}+\sqrt{{\left(x+\frac{a}{2}\right)}^{2}+{\left(y-\frac{b}{2}\right)}^{2}+z^{2}}}{y-\frac{b}{2}+\sqrt{{\left(x-\frac{a}{2}\right)}^{2}+{\left(y-\frac{b}{2}\right)}^{2}+z^{2}}}+\ln\frac{y+\frac{b}{2}+\sqrt{{\left(x-\frac{a}{2}\right)}^{2}+{\left(y+\frac{b}{2}\right)}^{2}+z^{2}}}{y+\frac{b}{2}+\sqrt{{\left(x+\frac{a}{2}\right)}^{2}+{\left(y+\frac{b}{2}\right)}^{2}+z^{2}}}\right]\;$$

(2) $$B_{y+}=\frac{B_{r}}{4\pi }\left[\ln\frac{x-\frac{a}{2}+\sqrt{{\left(x-\frac{a}{2}\right)}^{2}+{\left(y+\frac{b}{2}\right)}^{2}+z^{2}}}{x-\frac{a}{2}+\sqrt{{\left(x-\frac{a}{2}\right)}^{2}+{\left(y-\frac{b}{2}\right)}^{2}+z^{2}}}+\ln\frac{x+\frac{a}{2}+\sqrt{{\left(x+\frac{a}{2}\right)}^{2}+{\left(y-\frac{b}{2}\right)}^{2}+z^{2}}}{x+\frac{a}{2}+\sqrt{{\left(x+\frac{a}{2}\right)}^{2}+{\left(y+\frac{b}{2}\right)}^{2}+z^{2}}}\right]\;$$

$$B_{z+}=\frac{B_{r}}{4\pi }\left[\arcsin\frac{\left(x-\frac{a}{2})(y-\frac{b}{2}\right)}{\sqrt{{\left(x-\frac{a}{2}\right)}^{2}+z^{2}}\sqrt{{\left(y-\frac{b}{2}\right)}^{2}+z^{2}}}-\arcsin\frac{\left(x-\frac{a}{2})(y+\frac{b}{2}\right)}{\sqrt{{\left(x-\frac{a}{2}\right)}^{2}+z^{2}}\sqrt{{\left(y+\frac{b}{2}\right)}^{2}+z^{2}}}\right]$$

(3) $$-\frac{B_{r}}{4\pi }\left[\arcsin\frac{\left(x+\frac{a}{2})(y-\frac{b}{2}\right)}{\sqrt{{\left(x+\frac{a}{2}\right)}^{2}+z^{2}}\sqrt{{\left(y-\frac{b}{2}\right)}^{2}+z^{2}}}-\arcsin\frac{\left(x+\frac{a}{2})(y+\frac{b}{2}\right)}{\sqrt{{\left(x+\frac{a}{2}\right)}^{2}+z^{2}}\sqrt{{\left(y+\frac{b}{2}\right)}^{2}+z^{2}}}\right]$$

Similarly, the three components *B*_x−_, *B*_y−_, and *B*_z−_ originating from negative charge are given by the following:

(4) $$B_{x-}=\frac{B_{r}}{4\pi }\left[\ln\frac{y-\frac{b}{2}+\sqrt{{\left(x+\frac{a}{2}\right)}^{2}+{\left(y-\frac{b}{2}\right)}^{2}+{\left(z+h\right)}^{2}}}{y-\frac{b}{2}+\sqrt{{\left(x-\frac{a}{2}\right)}^{2}+{\left(y-\frac{b}{2}\right)}^{2}+{\left(z+h\right)}^{2}}}+\ln\frac{y+\frac{b}{2}+\sqrt{{\left(x-\frac{a}{2}\right)}^{2}+{\left(y+\frac{b}{2}\right)}^{2}+{\left(z+h\right)}^{2}}}{y+\frac{b}{2}+\sqrt{{\left(x+\frac{a}{2}\right)}^{2}+{\left(y+\frac{b}{2}\right)}^{2}+{\left(z+h\right)}^{2}}}\right]\;$$

(5) $$B_{y-}=\frac{B_{r}}{4\pi }\left[\ln\frac{x-\frac{a}{2}+\sqrt{{\left(x-\frac{a}{2}\right)}^{2}+{\left(y+\frac{b}{2}\right)}^{2}+{\left(z+h\right)}^{2}}}{x-\frac{a}{2}+\sqrt{{\left(x-\frac{a}{2}\right)}^{2}+{\left(y-\frac{b}{2}\right)}^{2}+{\left(z+h\right)}^{2}}}+\ln\frac{x+\frac{a}{2}+\sqrt{{\left(x+\frac{a}{2}\right)}^{2}+{\left(y-\frac{b}{2}\right)}^{2}+{\left(z+h\right)}^{2}}}{x+\frac{a}{2}+\sqrt{{\left(x+\frac{a}{2}\right)}^{2}+{\left(y+\frac{b}{2}\right)}^{2}+{\left(z+h\right)}^{2}}}\right]\;$$

$$B_{z-}=\frac{B_{r}}{4\pi }\left[\arcsin\frac{\left(x-\frac{a}{2})(y-\frac{b}{2}\right)}{\sqrt{{\left(x-\frac{a}{2}\right)}^{2}+{\left(z+h\right)}^{2}}\sqrt{{\left(y-\frac{b}{2}\right)}^{2}+{\left(z+h\right)}^{2}}}-\arcsin\frac{\left(x-\frac{a}{2})(y+\frac{b}{2}\right)}{\sqrt{{\left(x-\frac{a}{2}\right)}^{2}+{\left(z+h\right)}^{2}}\sqrt{{\left(y+\frac{b}{2}\right)}^{2}+{\left(z+h\right)}^{2}}}\right]$$

(6) $$-\frac{B_{r}}{4\pi }\left[\arcsin\frac{\left(x+\frac{a}{2})(y-\frac{b}{2}\right)}{\sqrt{{\left(x+\frac{a}{2}\right)}^{2}+{\left(z+h\right)}^{2}}\sqrt{{\left(y-\frac{b}{2}\right)}^{2}+{\left(z+h\right)}^{2}}}-\arcsin\frac{\left(x+\frac{a}{2})(y+\frac{b}{2}\right)}{\sqrt{{\left(x+\frac{a}{2}\right)}^{2}+{\left(z+h\right)}^{2}}\sqrt{{\left(y+\frac{b}{2}\right)}^{2}+{\left(z+h\right)}^{2}}}\right]\;$$

The magnet material utilized in the magnetic grating is ferrite powder, with residual magnetic flux density *B_r_* = 0.255 T and permeability *μ_r_* = 1.05. The magnetic grating comprised of the magnet, adhesion tape, and cover tape is flexible and thin, which is especially suitable for compact zoom lens systems. The total components of the magnetic flux density can be determined through the following equations: *B_x_* = *B_x_*_+_ − *B_x_*_−_, *B_y_* = *B_y_*_+_ − *B_y_*_−_, and *B_z_* = *B_z_*_+_ − *B_z_*_−_. This computational approach is equally applicable to magnets polarized in the downward direction ↓. Figure 2d illustrates the calculated *B*_z_ values from O_−_ to O_+_ for various *i* (where *i* = 1, 2, 3, 4, 5, 6) at a specified air gap *δ*_S_ = 0.15 mm (along the z-direction) and y = 0. The analysis reveals that the central magnets (*i* = 1) exert the most significant influence on the magnetic field distribution. In contrast, the magnets positioned at the extremities (*i* = 2, 3, …) exhibit a progressively diminishing contribution. As *i* increases, *B*_z_ demonstrates a convergent trend, stabilizing at *i* = 6. Consequently, the total length of the magnetic grating (40.8 mm) exceeds the travel distance of the zoom group (35.4 mm) by a margin that accommodates at least six pairs of magnets. This design ensures adequate magnetic field coverage and stability across the operational range.

### 3.2. Results Comparison Between FEM and EMC

**Figure 3:**
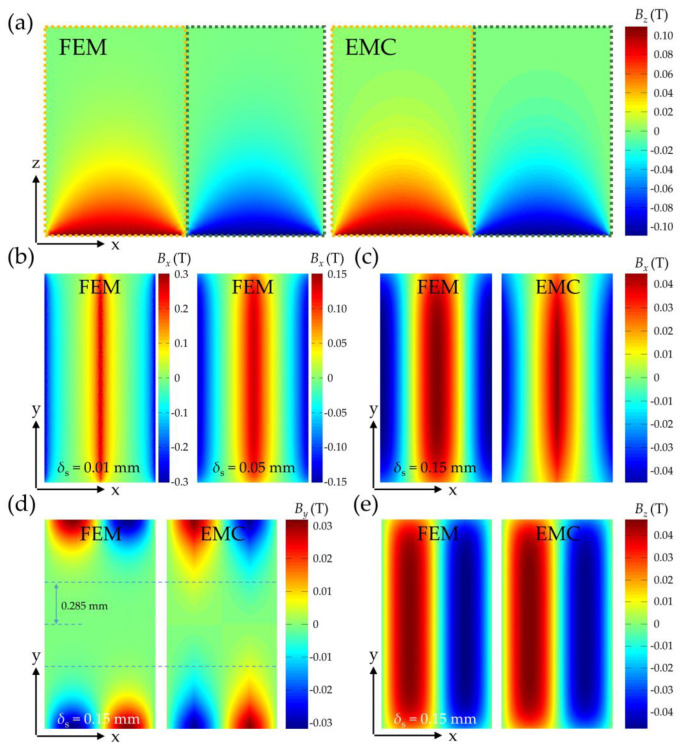
Results comparison between FEM and EMC methods. (**a**) *B_z_* at *x*–*z* plane (−0.4 ≤ *x* ≤ 0.4, *y* = 0, 0 ≤ *z* ≤ 0.6, unit: mm). (**b**) *B_x_* (FEM) at *x*–*y* plane (*δ*_S_ = 0.01 mm and 0.05 mm). (**c**) Results comparison for *B_x_* at *x*–*y* plane (*δ*_S_ = 0.15 mm). (**d**) Results comparison for *B_y_* at *x*–*y* plane (*δ*_S_ = 0.15 mm). (**e**) Results comparison for *B_z_* at *x*–*y* plane (*δ*_S_ = 0.15 mm). For (**b**–**e**): −0.4 ≤ *x* ≤ 0.4, −0.75 ≤ *y* ≤ 0.75, unit: mm.

Furthermore, the magnetic field distribution of the magnetic grating is numerically investigated using COMSOL Multiphysics 6.2 software based on FEM. Figure 3 presents a comparative analysis between FEM simulations and EMC analytical results. In Figure 3a, the dashed yellow (left) and green (right) boxes demarcate regions with ↑ and ↓ polarized magnets, respectively (consistent with Figure 3b–e). The *z*-component flux density (*B_z_*) distribution reveals distinct polarity patterns: Positive values (positive direction along the *z*-axis) and negative values (negative direction) correspond to the magnetization directions of adjacent magnets. Maximum *B_z_* values occur at magnet centers, while symmetry-induced null points emerge at domain boundaries. Axial field attenuation along the *z*-direction demonstrates an inverse relationship with vertical distance from the magnet surface.

In contrast, for *B_x_* in the *x*–*y* plane, field intensification occurs at magnetic domain boundaries with central null regions, as illustrated in Figure 3b,c. At *δ*_S_ = 0.01 mm, *B_x_* is predominantly concentrated near boundaries, exhibiting sharp gradient transitions. As *δ*_S_ increases to 0.05 mm and 0.15 mm, this transition becomes more gradual, with the performance that red and blue regions (|*B_x_*| > 0.8 *B_x_*_max_) expand from the boundaries toward the center. Due to symmetry, *B*_y_ equals zero along the centerline of the magnetic grating in the *x*-axis, which corresponds to the movement trajectory of the TMR sensor, as shown in Figure 3d. To reduce the influence on the TMR sensor signal and ensure its sinusoidal characteristic, the constraint is *B_y_* < 5 mT, and the corresponding lateral offset is less than 0.285 mm according to the computation. In addition, the flux density component perpendicular to the top surface of magnetic grating affects the signal hardly, and thus there is nearly no confinement from *B*_z_.

**Figure 4:**
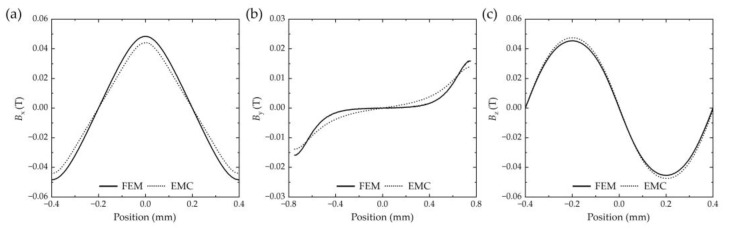
Results comparison between FEM and EMC methods. (**a**) *B_x_* (−0.4 ≤ *x* ≤ 0.4, *y* = 0, *z* = 0.15). (**b**) *B_y_* (−0.8 ≤ *y* ≤ 0.8, *x* = −0.2, *z* = 0.15). (**c**) *B_z_* (−0.4 ≤ *x* ≤ 0.4, y = 0, z = 0.15). unit: mm.

Figure 4 shows 2D plots for *B_x_*, *B_y_,* and *B_z_* between FEM and EMC methods from Figure 3c–e. The Pearson correlation coefficients r for *B_x_*, *B_y_,* and *B_z_* between the two methods are 0.9642, 0.9210, and 0.9789, respectively. The EMC solutions show strong congruence with FEM results across all parametric conditions. This verification confirms the accuracy of analytical computation of magnetic field distribution for magnetic grating, particularly in determining the optimal air gap *δ*_S_.

### 3.3. Analysis of Optimal Air Gap δ_S_

The *B*_x_ distribution in the magnetic grating is of paramount importance, as the TMR sensor directly detects *B_x_* parallel to its motion direction. The amplitude of *B_x_* must fall within an appropriate range: Excessive amplitudes may lead to signal clipping, while insufficient amplitudes result in poor detection sensitivity and a low signal-to-noise ratio. Furthermore, the *B*_x_ distribution should exhibit a high-quality sinusoidal profile to generate sinusoidal waveforms, thereby minimizing signal processing errors and reducing correction complexity. Consequently, determining the optimal air gap *δ*_S_ is critical to ensure both proper amplitude and sinusoidal distribution.

**Figure 5:**
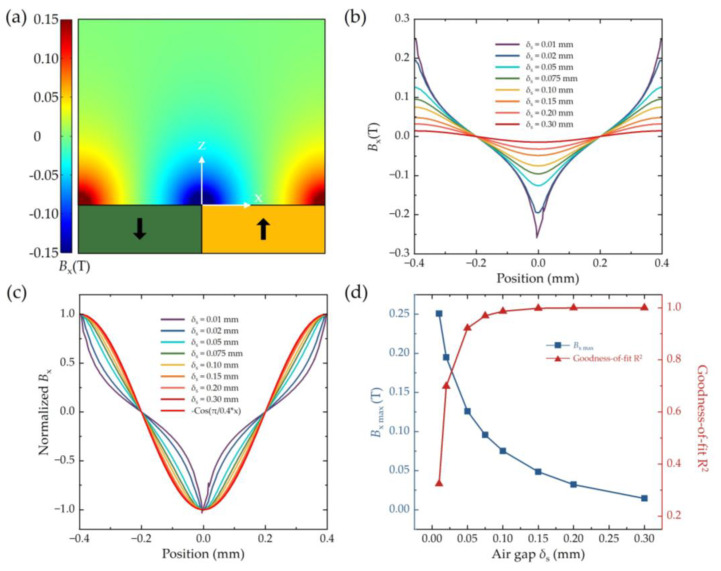
*B*_x_ calculated by FEM with different air gap *δ*_S_. (**a**) *B_x_* at *x*–*z* plane (−0.4 ≤ *x* ≤ 0.4, y = 0, 0 ≤ *z* ≤ 0.6, unit: mm). White arrows represent x-axis and z-axis. (**b**) *B_x_*–*x* position when *δ*_S_ = 0.01, 0.02, 0.05, 0.075, 0.10, 0.15, 0.20, and 0.30 mm. (**c**) Comparison between normalized *B_x_* at different *δ*_S_ with cosine distribution −Cos(π/0.4⋅*x*). (**d**) Peak value of *B*_x max_ and goodness-of-fit R^2^ between normalized *B_x_* and −Cos(π/0.4⋅*x*) for different *δ*_S_.

Figure 5 presents the FEM simulations of *B_x_* in the *x*–*z* plane (*y* = 0) for varying air gaps *δ*_S_. Similar to the *B_z_* results in Figure 3a, *B_x_* exhibits a periodic distribution along the *x*–direction with a period of 0.8 mm and axial field attenuation along the *z*-direction, as illustrated in Figure 5a. Two-dimensional cross-sectional plots along the *x*-direction for different air gaps (*δ*_S_ = 0.01, 0.02, 0.05, 0.075, 0.10, 0.15, 0.20, and 0.30 mm) are shown in Figure 5b, represented by rainbow-colored lines. At *δ*_S_ = 0.01 mm, cusp points appear at positions corresponding to the maximum *B_x_* amplitude, with a peak value of 251.1 mT, which is excessively large and detrimental to signal acquisition. As *δ*_S_ increases, the plots become smoother, and the cusp points gradually vanish. To analyze the waveform changes, the plots are normalized to their peak values, as depicted in Figure 5c. With increasing *δ*_S_, the normalized *B_x_* converges to −cos(π/0.4⋅*x*) (red line), indicating that the sensor must maintain a certain distance from the magnetic grating to achieve a high-quality sinusoidal distribution.

To quantify the sinusoidal characteristics and total harmonic distortion, the normalized *B*_x_ profiles are fitted to −cos(π/0.4⋅*x*). The peak values *B_x_*_max_ and goodness-of-fit R^2^ for different *δ*_S_ are summarized in Figure 5d. Here, the pair [*B_x_*_max_, R^2^] describes the *B*_x_ distribution conditions for each *δ*_S_. The R^2^ value increases rapidly from 0.3244 (*δ*_S_ = 0.01 mm) to 0.9216 (*δ*_S_ = 0.05 mm) and asymptotically approaches 1 as *δ*_S_ increases further. For the TMR sensor, the dynamic range of the magnetic field is about 20 mT to 120 mT. In consideration of the working range, the appropriate air gap range is determined to be 0.05 mm ≤ *δ*_S_ ≤ 0.20 mm, corresponding to [126.0 mT, 0.9216] to [32.1 mT, 0.9995]. The optimal air gap is identified as *δ*_S_ = 0.15 mm, with [48.5 mT, 0.9977] in ideal conditions without any interference. In addition, it is noteworthy that the air gap refers to the distance between the surface of the sensor package and the magnetic grating. Based on these analyses, the TMR sensor in the positioning system is designed to be positioned 0.15 mm above the magnetic grating.

### 3.4. Simplified Fitting Model

From Figure 5d, the peak value *B*_x max_ exhibits an exponential decay trend with increasing *δ*_S_, while the normalized *B*_x_ distribution converges to a cosine function for *δ*_S_ ≥ 0.05 mm. Based on these observations, a simplified fitting model for *B_x_* distribution within the range 0.05 mm ≤ *δ*_S_ ≤ 0.30 mm is proposed. This model combines an exponential decay function with a negative cosine function, capturing both the amplitude attenuation and the periodic spatial distribution. The resulting Expdec*Cos fitting model is expressed as follows:

(7) $$B_{x}=-0.19678\;\times \;e^{-\frac{\delta_{S}}{0.1072}}\;\times \;\cos(\pi /0.4\text{·}x)\;(T)\;$$

**Figure 6:**
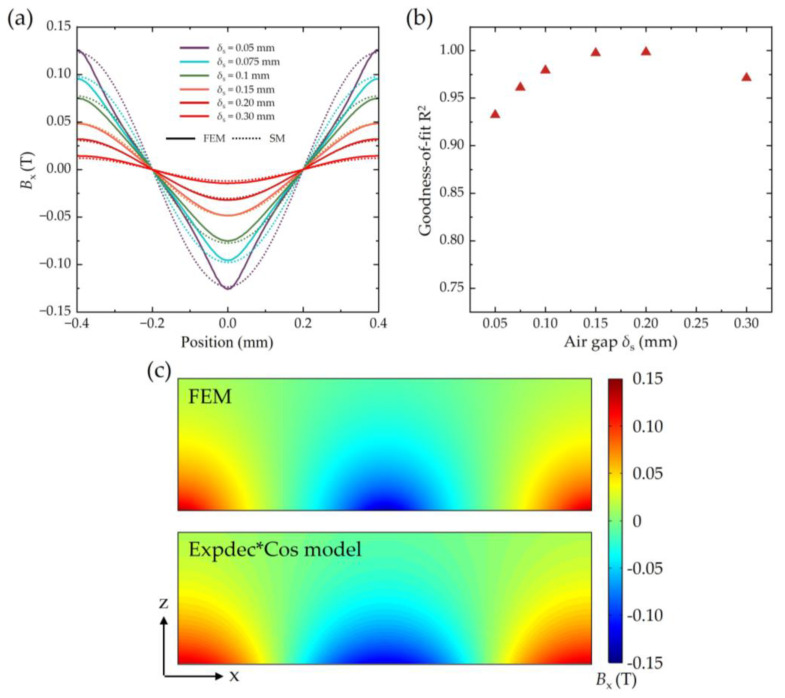
Results comparison between FEM and Expdec*Cos fitting model. (**a**) *B_x_*–position *x* when *δ*_S_ = 0.05, 0.075, 0.10, 0.15, 0.20, and 0.30 mm. “SM” represents “simplified model”. (**b**) Goodness-of-fit R^2^ for *B_x_* between fitting model and FEM results for different *δ*_S_. (**c**) *B_x_* calculated by FEM and simplified fitting model at *x*–*z* plane (−0.4 ≤ *x* ≤ 0.4, *y* = 0, 0 ≤ *z* ≤ 0.3, unit: mm).

Figure 6 shows a comparison between the FEM results and the simplified model predictions using Equation (7). As shown in Figure 6b, the goodness-of-fit R^2^ exceeds 0.93, demonstrating strong agreement between the simplified model and FEM simulations. This simplified model significantly reduces computational complexity while maintaining high accuracy, offering a practical tool for similar analytical computations of magnetic field distributions in related applications.

## 4. FEM Simulations of Magnetic Field Distribution for VCM and Measured Displacement

**Figure 7:**
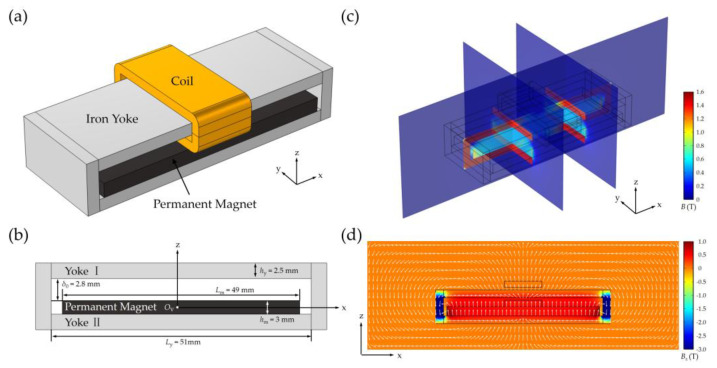
Schematic of VCM and corresponding magnetic flux density calculated by FEM. (**a**) Schematic of VCM comprised of coil, iron yoke, and permanent magnet. (**b**) Schematic of VCM (hidden coil) in the cross-sectional view. (**c**) Magnetic flux density *B* distribution for VCM. (**d**) *B_z_* at *x*–*z* plane (−50 ≤ *x* ≤ 50, y = 0, −12.5 ≤ *z* ≤ 22.5, unit: mm).

As described above, these results confirm that the optimal air gap between the sensor and magnetic grating is identified in ideal conditions without any interference. In this section, we examine the magnetic interference of VCM on signal output. The zoom optical system utilizes VCMs to actuate both the moving lens group and the TMR sensor. As illustrated in Figure 7a, the VCM configuration employs a 45SH NdFeB permanent magnet (Senyang Co., Ltd., Ganzhou, China) (stator) and a copper coil (mover), with the coordinate system origin aligned to the magnet’s geometric center *O*_V_, as shown in Figure 7b. The dimensions of the NdFeB magnet are as follows: length *L*_m_ = 49 mm, width *W*_m_ = 20 mm, and height *h*_m_ = 3 mm, with a residual magnetic flux density of 1.32 T [32]. The extended planar geometry of the magnet ($L_{m},\;W_{m}\;\gg \;h_{m}$) establishes a quasi-uniform axial magnetic field (*B* ∥ *z*) within the air gap, while the closed yokes enhance flux confinement and circuit permeability. For yokes, the dimensions are as follows: length *L*_m_ = 51 mm, width *W*_m_ = 20 mm, height *h*_y_ = 2.5 mm, and air gap *δ*_0_ = 2.8 mm. When the coil is energized, the wires that pass through the air gap experience Lorentz force that causes the coil, together with the lens group, to take linear motion. The number of turns of the coil is *N* = 400, and the current is *I* = 1.55 A. As the VCM operates based on electromagnetic force, it inevitably produces an external magnetic field, which may interfere with the TMR sensor. Specifically, the magnetic flux leakage from the magnet and electromagnetic field generated by the coil must be carefully considered, particularly the former. Therefore, it is essential to analyze the VCM’s magnetic field to identify an external region with relatively weak magnetic interference. This analysis is critical for determining the optimal relative positioning between the TMR sensor (*O*_S_) and VCM (*O*_V_) to minimize interference effects.

Figure 7c presents FEM simulations of magnetic flux density *B* distribution for VCM. The magnetic circuit is mainly confined in yokes and air gaps, while the flux density generated by the energizing coil is negligible compared to the magnetic field from the magnet. Because the relative permeability of copper is nearly 1 (equivalent to air in magnetics), the shape of the coil affects little flux density distribution. The boundaries that form the top and sides of the magnet are bordered by yokes with large permeability, and consequently, the tangential component vanishes along these boundaries. As shown in Figure 7d, the magnet polarized along the positive *z*-axis mainly generates a quasi-uniform axial magnetic field in the air gap with flux confinement by yoke I, while white arrows represent directions of the magnetic flux density. The field uniformity metric ((*B_z_*_max_ − *B_z_*_min_)/*B_z_*_mean_) is less than 14% across the central 80% of the air gap, validating the effectiveness of the yoke design in maintaining spatial field consistency. Compared to yoke-free configurations, iron yokes achieve a 29% improvement in field uniformity and a 213% enhancement in flux density.

**Figure 8:**
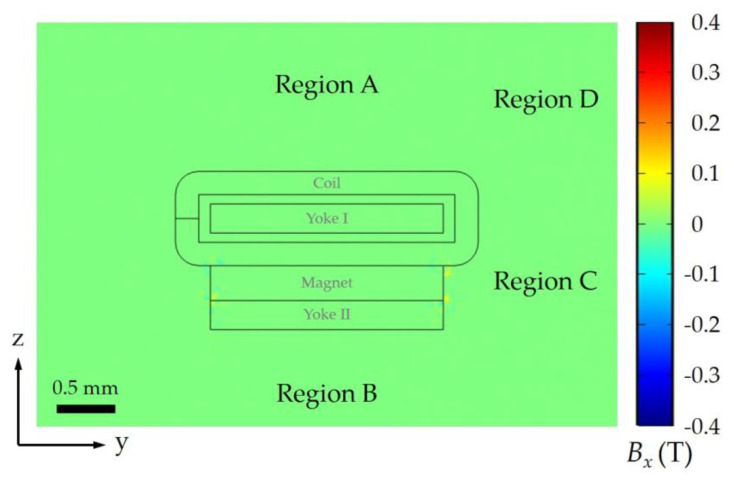
*B_x_* at *y*–*z* plane (−25 ≤ *y* ≤ 25, *x* = 0, −12.5 ≤ *z* ≤ 22.5, unit: mm) for VCM.

**Figure 9:**
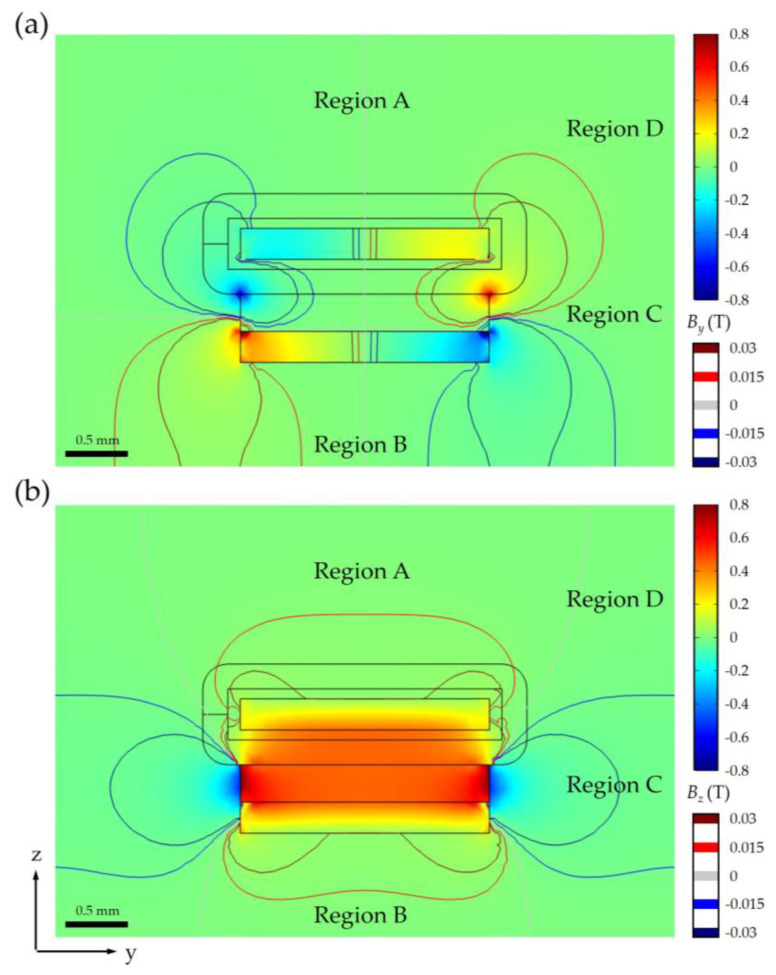
Magnetic flux density distribution (**a**) *B_y_* and (**b**) *B_z_* at *y*–*z* plane (−25 ≤ *y* ≤ 25, *x* = 0, −12.5 ≤ *z* ≤ 22.5, unit: mm) for VCM.

As the TMR sensor is mounted on the moving group, the direction of periodically arranged magnets of the magnetic grating is parallel to the direction of length for the VCM (i.e., along the *x*-axis) of necessity. Consequently, the remaining critical design considerations are determining the orientation of the top surface of magnetic grating and the relative positioning between the sensor and VCM. To address these aspects, a detailed analysis of the magnetic flux density distribution in the *y*–*z* plane is essential. FEM simulations were conducted to characterize this distribution, with the results presented in Figure 8 and Figure 9. These simulations provide critical insights into the spatial field variations, enabling the optimization of sensor placement and grating orientation to minimize interference and ensure accurate signal detection.

The potential positioning of the TMR sensor is categorized into four regions (A, B, C, and D) external to the VCM, corresponding to the top, bottom, side, and upper side. Given the sensor’s trajectory along the *x*-axis, *B_x_* is the primary detection parameter, and its distribution significantly influences signal integrity. Due to the presence of bilateral yokes at both ends of the permanent magnet along the *x*-axis, the *B_x_* distribution is nearly negligible across most of the *y*–*z* plane. However, *B*_x_ leakage is only observed in Region C, where the absence of yokes results in unconfined magnetic fields. Consequently, Region C is excluded to minimize interference effects on the sensor.

The *B_y_* primarily concentrates on the four corners of the magnet in the *y*–*z* plane, as illustrated in Figure 9a. The isolines reveal an antisymmetric *B_y_* distribution about the plane *y* = 0, which coincides with the *B_y_* = 0 isoline. Although the magnet’s mounting on yoke II breaks the symmetry along the z-direction, the *B_y_* = 0 isoline nearly aligns with the plane *z* = 0. While Regions A and B, where *B_y_* = 0, appear suitable for sensor placement, practical constraints limit their feasibility. Specifically, the moving group is fixed to the top surface of the coil, with optical lenses occupying Region A to maintain optical axis consistency. Additionally, the energized coil generates significant heat, leading to increased copper resistance and potential magnet demagnetization. To enhance thermal dissipation, the bottom surface of yoke II is exposed to ambient air, rendering Regions A and B unsuitable due to these external factors. Thus, alternative regions must be considered to optimize sensor placement while mitigating thermal and magnetic interference.

As illustrated in Figure 9b, *B_z_* distribution exhibits symmetry about the plane *y* = 0, with significant magnetic flux leakage observed in Region C. Consequently, Region D emerges as the optimal location for sensor placement, with the sensor position *O*_S_ ideally positioned as close as possible to the *B_z_* = 0 isoline to minimize interference. Within Region D, the magnitude of *B_y_* generally exceeds that of *B_z_*, necessitating the alignment of the magnetic grating’s top surface with the *x*–*z* plane of the VCM to mitigate the interference effects of the relatively strong *B_y_* component.

While theoretical analysis suggests that magnetic interference decreases with increasing distance from the VCM, practical constraints imposed by the overall dimensions of the zoom optical system and the geometry of the lens group flange limit the feasible placement of *O*_S_. After comprehensive consideration of these factors, *O*_S_ is designed to be located in the upper side region outside the VCM, with coordinates offset by 24 mm in the y-direction and 20 mm in the z-direction relative to *O*_V_. This configuration achieves an effective balance between minimizing magnetic interference and accommodating the mechanical and optical design requirements of the system.

**Figure 10:**
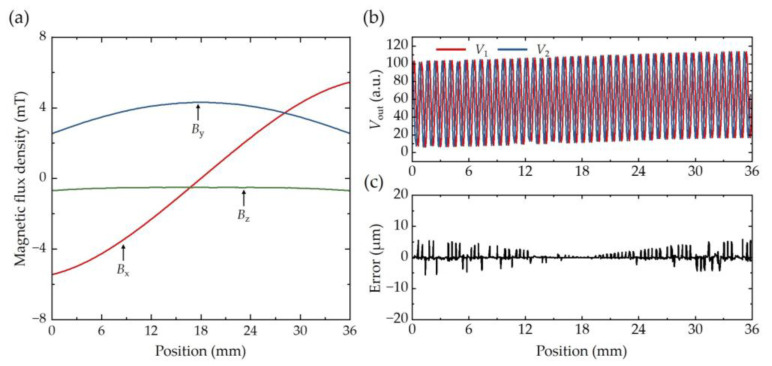
Magnetic interference from VCM and position error. (**a**) Magnetic flux density components at trajectory of sensor. (**b**) Signals output calculation of TMR sensor under magnetic interference from the VCM. (**c**) Calculation of position error caused by magnetic interference.

To evaluate the impact of magnetic interference on positioning accuracy, an analysis of position errors is essential, as it provides critical insights for magnetic shielding design and subsequent signal correction. Figure 10a summarizes the magnetic flux density components (*B_x_*, *B_y_*, and *B_z_*) along the sensor’s trajectory. The most influential component, *B_x_*, exhibits an antisymmetric distribution about the midpoint (located at 18 mm), with its magnitude increasing gradually from the midpoint toward the ends, reaching a maximum value of 5.4 mT. In contrast, *B_y_* and *B_z_* display opposing symmetry and variation trends, with maximum values of 4.3 mT and 0.5 mT, respectively.

By combining the periodic magnetic flux density distribution of magnetic grating with the magnetic interference from VCM, the resulting sensor output signals are calculated and depicted in Figure 10b. The interference causes deviations from the ideal sinusoidal and cosine waveforms. The position error, derived by comparing the affected signals with their standard counterparts, is generally less than 5 μm, as shown in Figure 10c. The minimum position error occurs at the midpoint of the trajectory, corresponding to the region of minimal magnetic interference, while the maximum errors are observed at both ends.

**Figure 11:**
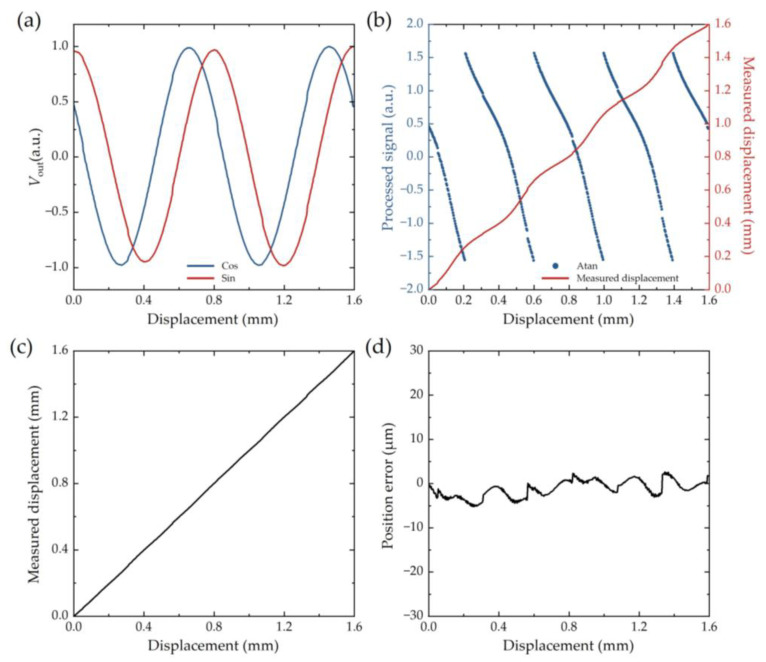
Signal processing. (**a**) Measured original signals of TMR sensor. (**b**) Processed signals and corresponding measured displacement. (**c**) Measured displacement after correction. (**d**) Position error.

Figure 11 presents the processing of measured original signals of the TMR sensor. Measured original signals are Sine and Cosine waveforms with a period of 0.8 mm. Besides magnetic interference, deviations from the ideal waveforms may originate from inhomogeneous magnetization of the magnetic grating, uneven air gap, and assembly alignment error. After Atan calculation, the measured displacement still exhibits an approximate periodic Cosine function of actual displacement with a period of 0.4 mm (see red line in Figure 11b), which should be corrected to be a linear function, as shown in Figure 11c. The position error is less than 5 μm, which is shown in Figure 11d.

**Figure 12:**
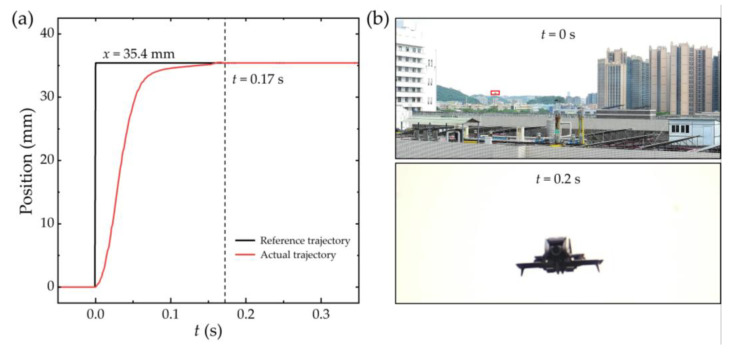
Control performance of moving group and photographs captured by rapid zoom lens. (**a**) Actual trajectory and reference trajectory of moving group. (**b**) Photographs captured by a 40× rapid zoom optical system with VCMs.

Utilizing the above-designed positioning system as position feedback of moving groups, the closed-loop control system is achieved with a photo-interrupter sensor (RPI-222, ROHM Co., Ltd., Kyoto, Japan) for absolute positioning for a 40× zoom optical system. The moving group is driven by VCM and controlled in linear motion with a stroke of 35.4 mm, and it costs about 0.17 s for the moving group to reach the destination, as shown by the actual trajectory in Figure 12a. Consequently, the zooming time from the short-focus state to the long-focus state is within 0.2 s for the rapid zoom optical system with VCMs. At *t* = 0 s, a security camera in a wide-area surveillance state captures a moving drone, and high-resolution identification is acquired fleetly at *t* = 0.2 s, as illustrated in Figure 12b. Rapid zoom optical lenses with VCMs show great potential in security surveillance applications.

## 5. Conclusions

In conclusion, this study systematically analyzes a high-precision positioning system comprising a TMR sensor and magnetic grating for high-magnification rapid zoom optical systems integrated with VCMs through numerical computations of magnetic field distributions. The EMC method demonstrates excellent agreement with FEM simulations, validating the accuracy of analytical computation of magnetic field distribution for magnetic grating. Through comprehensive optimization considering both flux density amplitude and waveform integrity, the optimal air gap between the sensor and magnetic grating is determined to be *δ*_S_ = 0.15 mm. A simplified Expdec*Cos fitting model is further proposed, significantly reducing computational complexity while maintaining high accuracy (R^2^ > 0.93). Given the electromagnetic nature of the VCM, magnetic interference from flux leakage is rigorously evaluated. The FEM results reveal that the magnetic grating’s top surface is perpendicular to the yoke-free side of the magnet (i.e., aligning with the *x*–*z* plane in VCM’s coordinate system) to minimize interference effects. Due to mechanical constraints imposed by the zoom optical system’s dimensions, the optimal sensor position *O*_S_ is strategically placed in the upper side region outside VCM, with offsets of 24 mm in the *y*-direction and 20 mm in the *z*-direction relative to VCM’s origin *O*_V_. Interference analysis confirms that the resulting position error remains below 5 μm, with maximum deviations occurring at the trajectory endpoints. After signal processing and correction, the measured displacement exhibits a linear relationship with the actual displacement, demonstrating the system’s robustness and precision. Based on the positioning system as the position feedback, closed-loop control is achieved for a 40× zoom optical system, and the zooming time from short-focus state to long-focus state is within 0.2 s. In the future, we will pay more attention to the interference effect of alternating current in coils and temperature variations to further enhance the position system’s performance. These findings provide a comprehensive framework for the design and optimization of magnetic positioning systems in high-performance optical applications.

## Data Availability

The raw data supporting the conclusions of this article will be made available by the authors upon request.

## References

[1] Gong J., Luo J. (2024). Rapid and Precise Zoom Lens Design Based on Voice Coil Motors with Tunnel Magnetoresistance Sensors. Appl. Sci..

[2] Zou T., Tang X., Song B., Wang J., Chen J. (2012). Robust Feedback Zoom Tracking for Digital Video Surveillance. Sensors.

[3] Liu S., Xie B., Yuan R., Zhang M., Xu J., Li L., Wang Q. (2023). Deep learning enables parallel camera with enhanced- resolution and computational zoom imaging. PhotoniX.

[4] Hu S., Shimasaki K., Jiang M., Senoo T., Ishii I. (2021). A Simultaneous Multi-Object Zooming System Using an Ultrafast Pan-Tilt Camera. IEEE. Sens. J..

[5] Jiang Z., Wang D., Zheng Y., Liu C., Wang Q. (2021). Continuous optical zoom microscopy imaging system based on liquid lenses. Opt. Exp..

[6] Zou Y., Chau F.S., Zhou G. (2017). Ultra-compact optical zoom endoscope using solid tunable lenses. Opt. Exp..

[7] Hou C., Ren Y., Tan Y., Xin Q., Zang Y. (2019). Ultra slim optical zoom system using Alvarez freeform lenses. IEEE Photonics J..

[8] Hsieh C., Liu C. (2020). Design of a voice coil motor actuator with L-Shape coils for optical zooming smartphone cameras. IEEE Access.

[9] Fleming A.J. (2013). A review of nanometer resolution position sensors: Operation and performance. Sens. Actuators A Phys..

[10] Costa C.F.R., Reis C.P. (2023). End-Point Position Estimation of a Soft Continuum Manipulator Using Embedded Linear Magnetic Encoders. Sensors.

[11] Lin H., Peng K., Chang J. (2024). Design and Evaluation of an Open-Structure Rotary Magnetic Encoder for Subaqueous Environments. IEEE Trans. Magn..

[12] Pan L., Xie Y., Li M., Bao X., Shang J., Shang J., Li R. (2023). Flexible Magnetic Sensors. Sensors.

[13] Zhao B., Wang L., Tan J. (2015). Design and Realization of a Three Degrees of Freedom Displacement Measurement System Composed of Hall Sensors Based on Magnetic Field Fitting by an Elliptic Function. Sensors.

[14] Yan S., Zhou Z., Yang Y., Leng Q., Zhao W. (2021). Developments and applications of tunneling magnetoresistance sensors. Tsinghua Sci. Technol..

[15] Lee C., Yen Y., Lai C. (2021). Alignment-Free Sensing Module for Absolute and Incremental Lines in Linear Positioning System Based on Tunneling-Magnetoresistance Sensors. Sensors.

[16] Wang X., Li W., Gong M., Wang J., Zhong Y., Ruan Y., Guo C., Xin C., Li M. (2022). High-precision micro-displacement sensor based on tunnel magneto-resistance effect. Sci. Rep..

[17] Silva J., Caetano D.M., Rabuske T., Cardoso S., Piedade M., Fernandes J.R. (2023). Integrated Circuit for Magnetic Encoder Sensing in TMR-Based Industrial Positioning System. IEEE T. Ind. Electron..

[18] Muna Y.B., Kuo C.C. (2020). Magnetic field distribution of magnetic encoder with TMR sensor using finite element analysis. Microsyst. Technol..

[19] Wang S., Peng D., Wu Z. (2019). Embedded Position Detection for Permanent Magnet Synchronous Motor with Built-In Magnets. IEEE. Sens. J..

[20] Lázár Z., Bidaux Y., Roost M., Close G.F. Model-Based Engineering of Magnetic Sensors. Proceedings of the 2019 16th International Conference on Synthesis, Modeling, Analysis and Simulation Methods and Applications to Circuit Design (SMACD).

[21] Dupre N., Bidaux Y., Dubrulle O., Close G.F. (2020). A Stray-Field-Immune Magnetic Displacement Sensor With 1% Accuracy. IEEE. Sens. J..

[22] Chen B., Chang J. (2019). Effect of Flattening Cracked Medium on Positioning Accuracy of a Linear Magnetic Encoder. IEEE Trans. Magn..

[23] Wang S., Feng B., Hu Y., Qiu G., Duan Z., Kang Y. (2023). Displacement measurement for ferromagnetic materials based on the double-layer parallel-cable-based probe. Sens. Actuators A Phys..

[24] Wang S., Wu Z., Peng D., Li W., Zheng Y. (2019). Embedded position estimation using tunnel magnetoresistance sensors for permanent magnet linear synchronous motor systems. Measurement.

[25] Luo C., Lin Z., Sun J. (2019). Design of linear voice coil motor with semi-closed structure. IET Electr. Power. App..

[26] Jiang Z., Park K., Kim J., Jiang Y., Xu D., Hwang S. (2020). Analysis and design of a new linear vibration motor used to reduce magnetic flux leakage in in-vehicle infotainment. Appl. Sci..

[27] Kang S., Jeong Y., Choi Y. (2023). Design of a finger-sized voice coil motor for high-speed scanners. Int. J. Precis. Eng. Man..

[28] Luo C., Sun J., Wang X., Shen Q. (2017). Design of voice coil motor with the forward winding for linear vibro-impact systems. IEEE Trans. Magn..

[29] Zhang Z., Luo M., Zhou H., Duan J. (2019). Design and analysis of a novel two-degree-of-freedom voice coil motor. IEEE-ASME T. Mech..

[30] Li J., Zheng Q., Song Y., Liu J. (1999). Computation of the Magnetic Field of Permanent Magnet with Equivlent Magnetic Charge Method. J. Yunnan Norm. Univ..

[31] Furlani E.P. (2001). Permanent Magnet and Electromechanical Devices: Materials, Analysis, and Applications.

[32] Dang Z., Qahouq J.A.A. Permanent magnet toroid power inductor with increased saturation current. Proceedings of the 2013 Twenty-Eighth Annual IEEE Applied Power Electronics Conference and Exposition (APEC).

